# A theoretical explanation for how a nutrition counseling and medically tailored meal delivery program benefitted participants living with lung cancer

**DOI:** 10.1007/s00520-024-08616-x

**Published:** 2024-06-13

**Authors:** Pamela Rothpletz-Puglia, Jade Smith, Chloe Pavuk, Jana Leotta, Kimberli Pike, Carolyn J. Presley, Jessica L. Krok-Schoen, Ashlea Braun, Mary Kathryn Cohen, Gail T. Rogers, Kenneth Kwan Ho Chui, Fang Fang Zhang, Colleen K. Spees

**Affiliations:** 1 Rutgers, The State University of New Jersey, School of Health Professions, New Brunswick, NJ USA; 2 School of Health and Rehabilitation Sciences, Division of Medical Dietetics, The Ohio State University, College of Medicine, Columbus, OH USA; 3 The James Comprehensive Cancer Center, Columbus, OH USA; 4 Division of Medical Oncology, Department of Medicine, The Ohio State University Comprehensive Cancer Center, Columbus, OH USA; 5 School of Health and Rehabilitation Sciences, Division of Health Sciences, The Ohio State University, College of Medicine, Columbus, OH USA; 6 TSET Health Promotion Research Center, Stephenson Cancer Center, University of Oklahoma Health Sciences, Tulsa, OK USA; 7 Friedman School of Nutrition Science and Policy, Tufts University, Boston, MA USA; 8 Department of Public Health and Community Medicine, Tufts University School of Medicine, Boston, MA USA

**Keywords:** Food is Medicine, Lung cancer, Nutrition intervention, Motivational interviewing, Nutrition security, Active coping, Post-traumatic growth, Medically tailored meals, Medical nutrition therapy, Quality of life

## Abstract

**Purpose:**

The purpose of this study was to assess participants’ perceptions and experiences while participating in a Food is Medicine medically tailored meal plus intensive nutrition counseling intervention to create a theoretical explanation about *how* the intervention worked.

**Methods:**

This interpretive qualitative study included the use of semi-structured interviews with active participants in a randomized controlled trial aimed at understanding how a medically tailored meal plus nutrition counseling intervention worked for vulnerable individuals with lung cancer treated at four cancer centers across the USA. During the 8-month long study, participants in the intervention arm were asked to be interviewed, which were recorded, transcribed verbatim, and analyzed using conventional content analysis with principles of grounded theory.

**Results:**

Twenty individuals participated. Data analysis resulted in a theoretical explanation of the intervention’s mechanism of action. The explanatory process includes three linked and propositional categories leading to patient resilience: engaging in treatment, adjusting to diagnosis, and active coping. The medically tailored meals plus nutrition counseling engaged participants throughout treatment, which helped participants adjust to their diagnosis, leading to active coping through intentional self-care, behavior change, and improved quality of life.

**Conclusions:**

These findings provide evidence that a Food is Medicine intervention may buffer some of the adversity related to the diagnosis of lung cancer and create a pathway for participants to experience post-traumatic growth, develop resilience, and change behaviors to actively cope with lung cancer. Medically tailored meals plus intensive nutrition counseling informed by motivational interviewing supported individuals’ adjustment to their diagnosis and resulted in perceived positive behavior change.

## Introduction

Lung cancer affects 1 in 15 men and 1 in 17 women annually and is the leading cause of all cancer-related deaths in the USA [1]. An estimated 80% of people undergoing treatment for lung cancer experience malnutrition, which is characterized by rapid, involuntary weight loss, muscle wasting, and decreased immune function [1, 2]. Individuals presenting with malnutrition often experience greater toxicities to cancer treatment, more frequent hospital admissions, extended length of hospital stays, lower quality of life, and increased mortality [1, 3]. Cancer-related malnutrition is often attributed to the metabolic burden of cancer and side effects of treatments which can lead to nutrition-impact symptoms including fatigue, anorexia, constipation, nausea, diarrhea, mucositis, and gastrointestinal distress [2]. The risk of experiencing malnutrition is compounded when the individual is from a medically underserved population (uninsured, rural resident, racial/ethnic minority, ≥65 years old, and/or low-income) and/or experiencing nutrition and/or food insecurity [4, 5].

Oncology Registered Dietitian Nutritionists (RDNs) are specifically trained to address nutrition impact symptoms and mitigate risks for malnutrition, but fewer than 60% of people undergoing treatment for cancer receive any nutrition services [3, 6]. Access to RDNs (“dietitians”) is limited in the USA due to inconsistent standardized malnutrition screening and lack of reimbursement for nutrition care from the Center for Medicaid and Medicare Services and other insurers [3, 7]. With limited insurance reimbursement available, there remains inadequate oncology dietitian staffing in outpatient cancer centers, where 90% of individuals affected by cancer receive treatment [3]. Indeed, the current ratio of dietitians to outpatients with cancer across the USA is one dietitian to every 2308 patients [3].

*Food is Medicine* efforts are underway to improve access to nutrition care and address the root causes of malnutrition among individuals with cancer [8]. Specifically, the provision of home-delivered medically tailored meals has been shown to reduce nutrition and food insecurity and improve quality of life among people receiving treatment for metastatic cancer [9, 10]. Likewise, intensive nutrition counseling, delivered by dietitians, has been successful in mitigating nutrition impact symptoms and reducing malnutrition risk and severity [6, 11]. However, findings employing dietitians are not consistently successful [12], which may be secondary to differences in the content of one-on-one interactions. For example, if dietitians primarily focus only on medical nutrition therapy centered on managing and/or alleviating symptoms, this may undermine the emotional aspects of diet and eating [13]. Alternatively, employing empathetic counseling approaches, such as motivational interviewing, may offer an advantage. Medical nutrition therapy enhanced via the use of motivational interviewing has been identified as an effective intervention for modifiable lifestyle behavior change among individuals experiencing cancer [14]; however, the experiences of patients receiving this unique treatment combination have not been explored. Motivational interviewing is an efficacious form of counseling that is centered on therapeutic empathy and focuses on honoring individual autonomy in pursuit of behavior change. It balances strong interpersonal skills (e.g., developing a strong patient/provider partnership) with tangible counselor behaviors (e.g., reflective listening) intended to cultivate an atmosphere of compassion and acceptance, while helping individuals overcome ambivalence towards behavior change [15, 16]. It has been found efficacious in various diet-related interventions; however, the mechanisms defining how and why MI works are largely based in substance use literature [17]. This enhanced counseling approach is a technique that provides a person-centered supportive environment conducive to mutual trust, engagement, and goal setting.

*NutriCare* is the first medically tailored meals plus intensive nutrition counseling intervention in the Food is Medicine space designed to fully integrate food and nutrition into oncology care for vulnerable patients with lung cancer. This multi-center randomized controlled clinical trial (NCT04986670) is provided throughout active cancer treatment and into post active treatment survivorship. Preliminary findings from this study documented improvements in diet quality, food security, and quality of life in the intervention group as compared to controls, and we wanted to understand how the NutriCare intervention worked to promote these outcomes [18, 19].

The research team developed a phase 2 qualitative study. The aim was to understand participants’ perceptions of the intervention to identify potential mechanisms, and then using these data to develop a theoretical explanation to guide future interventions. Theoretical explanations are based on patterned relationships between concepts that include propositional logic explaining why something may happen in a similar context [20].

## Methods

### Study design

This phase 2 qualitative inquiry was based in pragmatism where participant perceptions about useful aspects and applications of an intervention were generated from in-depth interviews [21]. Principles of grounded theory were implemented with conventional content analysis of the interview transcripts to generate categories (content analysis) [22] for theory about how the intervention worked (grounded theory) [23]. Grounded theory generates substantive theory. A substantive theory refers to an empirically based theory about a specific situation, and grounded theory methodology is generally atheoretical since the goal is to generate theory [17]. This pragmatic qualitative approach enabled the flexibility to incorporate principles of grounded theory using content analysis since content analysis and grounded theory are based on constructivism and are methodologically congruent [24].

### Ethical approval

The protocol was approved by the Institutional Review Boards of The Ohio State University (#2021E093) and Tufts University (#00002515) and adheres to the principles of the Declaration of Helsinki. All participants were informed of the purpose and procedures of the study. Verbal consent was obtained from all participants prior to data collection.

### Participants, intervention, and recruitment

Phase 1 of the NutriCare study enrolled 158 individuals recently diagnosed with lung cancer from four medical centers nationwide: Tufts Medical Center, MD Anderson Cancer Center, The Ohio State University Comprehensive Cancer Center, and Fox Chase Cancer Center. All participants met one or more of the following US Health Resources and Services Administration vulnerability criteria: uninsured, rural residency, racial/ethnic minority, ≥65 years old, and/or low income [4].

Within each site, patients were randomized into the NutriTool (enhanced control) or NutriCare (intervention) groups in a ratio of 1:1. Patients in the NutriTool arm received a printed copy of an evidence-based nutrition booklet for cancer survivors and monthly emails with basic nutrition information and recipes. Patients randomized to the NutriCare arm additionally received medically tailored meals (MTMs) delivered to their residence and remote nutrition counseling provided weekly by RDNs. The MTMs, provided by MANNA (https://mannapa.org/), were nutritionally tailored to meet the needs of cancer patients. The study dietitians prescribed weekly MTMs based upon nutrition impact symptoms from 11 different types of meals such as high calorie, high protein, mechanically soft, diabetic friendly, etc. NutriCare patients were provided three meals per day plus snacks for 7 days per week initially for 8 weeks followed by deliveries every other week for the next 8 weeks and concluding with delivery once a month for the final 8 weeks. Weekly remote nutrition counseling continued weekly for 32 weeks.

During week 8 of the 32-week intervention, the study dietitian assessed the participants’ willingness to participate in an interview with a graduate student researcher. This time frame corresponded with the end of the first and most intensive phase of the study. At this point in the study, NutriCare participants had received 6 to 8 weeks of both medically tailored meals plus intensive nutrition counseling. If a patient agreed to participate, the dietitian scheduled an interview between the participant and a trained graduate student researcher. A total of 20 participants on the NutriCare group consented and complete the interviews [25].

### Pre-interview training and development of the interview guide

Prior to beginning data collection, two graduate student researchers completed three 1-h qualitative research training sessions led by research team members with expertise in motivational interviewing (JS) and qualitative research (PRP). The interview guide was developed based on the preliminary findings from the NutriCare RCT. Each of the two graduate students (CP, JL) conducted a pilot semi-structured interview with a NutriCare participant and an experienced interviewer mentor (PRP). Pilot interviews were used pilot test and modify the interview guide and to provide the graduate students with feedback on interviewing techniques.

### Data collection

Semi-structured, telephone-based, audio-recorded interviews were conducted in English by graduate student researchers between August 2021 and February 2022. Probes within the interview guide (Table 1) allowed for flexibility in participant responses. Interviewers kept a reflexivity memo of thoughts, perceptions, and personal assumptions after each interview, and these experiences were discussed during regular team meetings.

**Table 1:** Interview guide

1. To get started, I would like to learn more about you. Please tell me about yourself and walk me through a typical day for you.	
2. How have your daily routines and plans changed since your diagnosis? **Probe:** How do you feel about these changes?	
3. Now, I want to ask you about the NutriCare program. What has this experience been like for you? **Probe:** How do you feel about the conversations with the dietitian **Probe:** Have the conversations affected other areas of your life/routine/wellness? **Probe:** Can you share your thoughts about the meals you’ve received? **Probe:** Has receiving meals affected other areas of your life/routine/wellness? **Probe:** Did you feel you could make the changes the nutritionist suggested? **Probe:** Do you mind giving me an example of a change you’ve made?	
4. How could we improve the Nutricare program?	
5. What advice would you share for other people living with lung cancer about how to adjust to it?	

### Data analysis

Interviews were audio-recorded, transcribed verbatim, and checked for accuracy to ensure an exact account of participant responses. ​Transcripts were anonymized and then analyzed using conventional content analysis and with principles of constructivist grounded theory [22, 23, 26]. The analysis began with five research team members each creating a conceptual memo for each of the first nine interview transcripts. The conceptual memos each team member created led to team discussions and consensus on initial concepts and the development of a preliminary codebook. The interview transcripts were then loaded into NVivo12 ^TM^ for first level, line-by-line coding (i.e., labeling concepts) by the graduate student researchers. The coding was assessed at >80% agreement among the two analysts. After consensus was reached on the discrepant coding, the graduate students double-coded 11 more transcripts. The research team met regularly to discuss and compare the codes to generate tentative categories of conceptually linked concepts. Once the team achieved consensus on the categories generated, the categories were compared within and between participant transcripts for identification of patterns, divergence, and linkages for development of a propositional logic to develop an explanatory process for the NutriCare intervention [27]. Recruitment, interviews, and analysis continued until data saturation was met, defined as the absence of new codes.

## Results

A total of *n* = 20 active NutriCare participants were interviewed between August 2021 through February 2022 and included in the data analysis (Table 2). Three additional participants consented to the study but did not answer the phone for their scheduled interview. Data saturation was determined after 17 interviews, but three additional interviews were conducted to ensure sufficient data for thorough descriptions to be applicable in other contexts (i.e., transferability) [28]. The duration of the interviews ranged from 14 to 64 min with an average interview length of 30 min.

**Table 2:** Participant characteristics (*n* = 20)

Mean age (years)	65.8 (9.5)
Age categories (years)	
35–<60	3 (15.0%)
61–<70	9 (45.0%)
70–<80	8 (40.0%)
Patient assigned sex at birth	
Male	7 (35.0%)
Female	13 (65.0%)
Race	
Non-Hispanic White	17 (85.0%)
Non-Hispanic Black	3 (15.0%)
Household income	
$15,000–$19,999	3 (15.0%)
$25,000–$34,999	6 (30.0%)
$45,000–$54,999	3 (15.0%)
$75,000+	6 (30.0%)
Don’t want to answer	2 (10.0%)
Health insurance	
Yes	20 (100.0%)
Medicare	
Yes	9 (45.0%)
No	10 (50.0%)
Don’t want to answer	1 (5.0%)
Mean Body Mass Index (BMI)	29.2 (7.4)
BMI categories	
Underweight (<18.5)	1 (5.0%)
Normal weight (18.5–24.9)	2 (10.0%)
Overweight (25.0–29.9)	10 (50.0%)
Obese (≥30)	7 (35.0%)
Have you smoked at least 100 cigarettes in your entire life?	
Yes	18 (90.0%)
No	2 (10.0%)
Number of comorbidities	
No comorbidity	2 (10.0%)
1–2 comorbidities	14 (70.0%)
3–4 comorbidities	4 (20.0%)
Histologic type of lung cancer at diagnosis	
Small cell	2 (10.0%)
Non-small cell	18 (90.0%)
Early or late-stage	
Early (Small cell limited/Non-small cell stage I–II)	3 (15.0%)
Late (Small cell extensive/Non-small cell stage III–IV)	17 (85.0%)
Recurrent/metastatic disease	
Yes	11 (55.0%)
No	9 (45.0%)
Eastern Cooperative Oncology Group (ECOG) Performance Status	
0	5 (25.0%)
1	13 (65.0%)
2	2 (10.0%)

### Participant demographics and clinical characteristics

Fifty-five percent of the interviewed participants had stage IV lung cancer (*n* = 11), six participants had stage III lung cancer, one participant had stage II, one participant had stage I, and one participant was classified as limited stage small cell lung cancer, in which malignancy was contained to one lung and treatable. Of the 20 participants, 65% were female (*n* = 13), 85% (*n* = 17) were white, and 55% (*n* = 11) were ≥65 years old. Forty percent (*n* = 8) of participants were rural residents, 100% (*n* = 20) had health insurance, 45% (*n* = 9) were on Medicare, 85% (*n* = 17) reported to be at or above 130% of the poverty line, and two participants declined to answer this question (Table 1).

### Explanatory theoretical process framework for the NutriCare intervention

We generated a substantive theory about how the NutriCare intervention worked. This explanatory process framework is composed of three propositional categories that we generated from the data to describe how the NutriCare intervention worked. Participants described how the NutriCare intervention works as follows:

1. **Engaging in treatment**: The medically tailored meals plus intensive nutrition counseling informed by motivational interviewing catalyzed participants’ engagement in their self-care because it was patient-centered, socially supportive, challenging, and empowering.
2. **Adjusting to diagnosis**: Engagement in the intervention helped participants adjust to their diagnosis because the meals were convenient and provided food and nutrition security. The support created the capacity for participants to adjust.
3. **Active coping with health behavior**: Participants’ adjustment to their diagnoses enabled learning, active coping, and post-traumatic growth during the intervention.

This process led to the overarching finding that the NutriCare intervention may have provided a pathway for participants to develop resilience for actively coping with lung cancer.

#### Engaging in treatment

Participants received and reported consuming weekly medically tailored meals and engaged in the intensive nutrition counseling. The drivers of engagement in the NutriCare intervention were related to participant’s perceptions of support, encouragement, empowerment, trust, being challenged, and feeling heard.

The weekly nutrition counseling with motivational interviewing engaged participants since they felt heard. Many participants mentioned how the dietitian listened:

“She listens. She listens to things I say. She's there when, you know — if there's something on my mind. She listens to — you know — things, or how I feel. And she tries to answer questions for me.” (O5)

In addition to active listening, participants described the nutrition counseling as personalized. For example, “She is trying to work with me and make suggestions within my stubbornness (laughs) — would be a way to say it. Yeah, I mean …I think [about] what she has shared with me, and what I've tried to…incorporate.” (O11)

Participants also described feeling encouraged by the dietitian during the nutrition counseling, and one participant stated, “She boosts my morale.”(F2) Another participant explained:

“At first, I thought, this is ridiculous to call and talk to me about food. I think she's really done some good by pushing me. She'll say okay, next, let's see if you can add a vegetable or two vegetables — so, she's challenged me. And I guess when somebody challenges me, I’ll take ‘em on it.” (M3)

This quote exemplifies how the dietitian challenged the participant to engage in care. The goal setting that often occurred also appeared to encourage participants. For example, “She had me drink those little shakes when I get nauseated and keeping something in my stomach. That was it. That was one of the goals. To keep something [in my stomach], eat little meals throughout the day which helped.”(M5)

The participants also discussed how the dietitian knew when to scale back on the goal setting as demonstrated by this quote, “She sets goals for me every week. We set them together actually. Like, on those weeks I wasn't feeling good after the radiation, I told her, [dietitian] I really don't have a goal this week, except for surviving and get through this. And she said, that's all we need.” (M3)

The dietitian provided tailored and person-centered care that engaged participants during all phases of their cancer treatment. For example:

“Yeah so [dietitian] provided lots of tips. I had 30 radiation treatments, and they burned my esophagus, and it has only been healed a few weeks. So you know, eating was hard for a long, long time and there were weeks that that I maybe ate 3 bites a day. It got pretty rough in the end, but when I started feeling better, you know hearing those times, you know encouraging me to eat soft foods and soup..” (O6)

The medically tailored meals provided a convenient resource to patients, making the collaborative nutrition counseling goals achievable. For example:

“I couldn't eat. So that's where MANNA came through for me”. They had certain things that they had pureed. They have many options for me….things that were more tolerable for me. And financially, that also helped me.” (O1)

Some participants provided feedback to improve the portions (*n* = 7), and types of foods (*n* = 11) in the meal deliveries, but all participants reported benefiting from the meal delivery. In addition to the financial and time benefits described, participants shared that the meals enabled them to focus more on and prioritize their nutrition status. One patient stated: “So NutriCare has set up a system of meals..... before I went on ..... NutriCare, I didn't eat all day. I just ate one meal at night.” (M6)

The participants reported engaging in the NutriCare intervention because they felt heard, supported, and encouraged during the intervention. This approach created patient and provider trust. For example, a participant stated:

“I made a commitment to [oncologist] that she would help me, get me well enough to see my grandson born, which has been seven months ago. Cause she'd work with me, and fight with me, and everything else..” (O2)

The participants’ engagement in the NutriCare intervention is attributable to the patient-provider relationships created during the tailored nutrition counseling and to the medically tailored meal delivery. The combination of nutrition counseling with motivational interviewing, perceived benefits, trust, and person-centered care empowered participants to engage in self-care in multiple ways, including consuming medically tailored meals despite not feeling well.

#### Adjustment to diagnosis

Most participants talked about the difficulty of living with lung cancer. For example, “I'm going through radiation right now and it's a bad thing, my esophagus’s not okay, and it really, it wears me out terribly I mean I'm just exhausted.” (F1) However, the participants perceived the NutriCare intervention as equipping them to handle their illness by providing tips and solutions that created the personal bandwidth to adjust to their lung cancer diagnosis. For instance participants talked about the benefits of meal delivery, including reduced stress related to meal preparation. One participant shared:

“I'm not able to, like, go out or cook and stuff. So, it’s good to know that there's something in the freezer that I can take out. So, I mean, I know this is going to sound really silly but I like the fact that I really don't have to think about food shopping.” (F1)

Participants talked about how the NutriCare intervention helped them when they were feeling unwell. One participant commented on how the NutriCare intervention helped her adjust by stating “those tips that she's giving you — been able to — that kinda helps your cooking process. Giving you some ideas of things to cook for yourself or kind of just help you cope with those really tough treatments and diagnosis and things like that.” (O6) Several participants discussed the specific benefits of pre- and probiotic foods and nutrition recommendations for helping them deal with and adjust to digestive issues. . For example:

“[NutriCare] has completely straightened out my old digestive system, and that was a miserable stage for me in the physical condition that I’m in. And I had wished I owned stock in Imodium^TM^ I was having to use it so much. So, I have none [diarrhea]. Absolutely none under your food and probiotic pill from [medical center] and [dietitian’s] list of 10 or 12 prebiotic foods to incorporate with that.” (M6)

Many (*n* = 15) of the participants’ comments related to learning new ideas and tools to help them adjust to their diagnosis:

“I could sort of put in my toolbox to get me through.” and “I really enjoyed the conversation with [dietitian]. She's given me really a lot of good ideas and some good recipes.” (F1)

The new ideas that participants discussed as helping them ranged from tangible nutrition tips to intentional practices for relaxation, stress relief, and physical activity patterns. The participants acknowledged that the health and wellness tips they received improved their quality of life. One participant stated, “I've had pretty good quality of life, you know, through the sickness. I mean, I'm stage 4, and uh you know, I've had a couple difficulties here and there, but for the most part I've got a good quality life. And I think a lot of that is due to my nutrition.” (O2)

Upon completion of the most intensive portion of the intervention (first 8 weeks of weekly meals plus weekly remote nutrition counseling), patients recognized the difference the intervention had made in their ability to improve their health and cope with their diagnosis. Moving them to share what a difference the NutriCare program could have had this opportunity been available earlier. For example, two patients shared:

“40, 50 years old they should be thinking about taking care of themselves I mean, I’ve learned that, that’s real. Nutrition is very important. Very important. You don’t realize. More than that, I mean if I had been doing this to begin with I might not have even wound up at the doctor. If you do your body right to start with, you might not have even wound up with this crap.” (O9)

In addition, to the recognition of the role nutrition plays for cancer prevention patients value the support medically tailored meals and counseling provide during treatment stating, “I wish I would have had this program when I was diagnosed the first time.” (M6)

#### Active coping with health behavior change for self-care

The NutriCare intervention supported participants to adjust to their diagnosis by helping them overcome nutrition and meal preparation challenges. Participants described overcoming some of the nutrition challenges of living with lung cancer by changing health behavior to actively cope and enact self-care. A participant stated:

“I want to prolong life, and if I was to do that, I'd have to be part of the work myself. I can't rely on drugs and treatments and everything else that I did... I got to know [dietitian] real well... Talking with her, she's like a godsend as far as, to help me out, give me different ideas when I was feeling sick and what to eat before chemo and after..” (O2)

The participants described making numerous dietary and lifestyle changes due to the NutriCare intervention. These behavior changes included drinking more water, eating more fruits, vegetables, and fiber, and eating less highly processed foods. One participant said:

“I no longer smoke anything. I try not to eat – very rarely do I eat red meat...and I try to make sure to eat fruits and vegetables every day..” (M2)

Many of the participants talked about how their dietary patterns are much healthier now. For example, a participant shared: “I mean I'm eating salads, I’m eating fruits. I’m eating, you know, I’m eating good food.” (M5)

The changes they made were incremental and sometimes described as difficult. For example, a participant stated: “So I figured I’d start learning to change. You know, so that's what I'm doing, and when you've ate one way for 60 some odd years. It's a challenge to change your way of thinking.” (M3) Some participants described how it was difficult to eat differently than the rest of the family. For example, “My husband is still eating southern comfort foods while I’m eating three [medically tailored] meals. And I stay on it religiously. I have stayed on it religiously.” (M6)

All the participants made dietary changes and often described how their eating patterns have substantially changed due to NutriCare. One participant said:

“At first it was difficult. I'm not going to say it wasn't because I was used to eating — maybe something for breakfast — a lot of times not and just eating a large supper. From being — like I said — working 8-10 hours a day, I just didn't eat. I didn’t eat well, I didn't eat three meals a day, and I didn't eat the proper food. I would wait until supper time, and then I’d just basically gorged myself. So, I'd be full through the next day. And the NutriCare, like I say, system working with [dietitian] and everything else has taught me that I have to have a minimum of three meals a day. And then even when I am sick, I just got over pneumonia and had radiation treatment and everything else. I might eat four or five meals a day — smaller meals.” (O2)

Another shared:

“What that's taught me though is that I've got nutrition throughout the day, and I have my energy throughout the day. And you know, it's taught me about eating well balanced food. Like right now — I used to eat a lot of white bread and stuff like that….If I do eat bread, I eat whole wheat or whole grain, but I don't eat that much bread anymore. I don't eat that much red meat anymore.” (O11)

Patients recognized both their own role in the intervention and the benefits provided to them through counseling.

One patient stated, “ So nutrition is very important it has to be part of your regular everyday life” indicating their understanding that they are responsible for their choices daily, while also expressing appreciation for the guidance provided through the nutrition counseling as stated here, “ Personally, I think every cancer patient .... should see a dietitian every time they see their doctor because the thing is, if we don't eat right, we’re not going to have the energy or the right nutrition for our body to fight anything.” O9

In addition to dietary changes, many participants also discussed increasing their physical activity, and time with family, friends, and pets. For example, “I guess with some encouragement from [dietitian]. I go to the Y every morning at eight and exercise in the pool. And then do yoga after that.” (M3)

#### Resilience

**Fig. 1:**
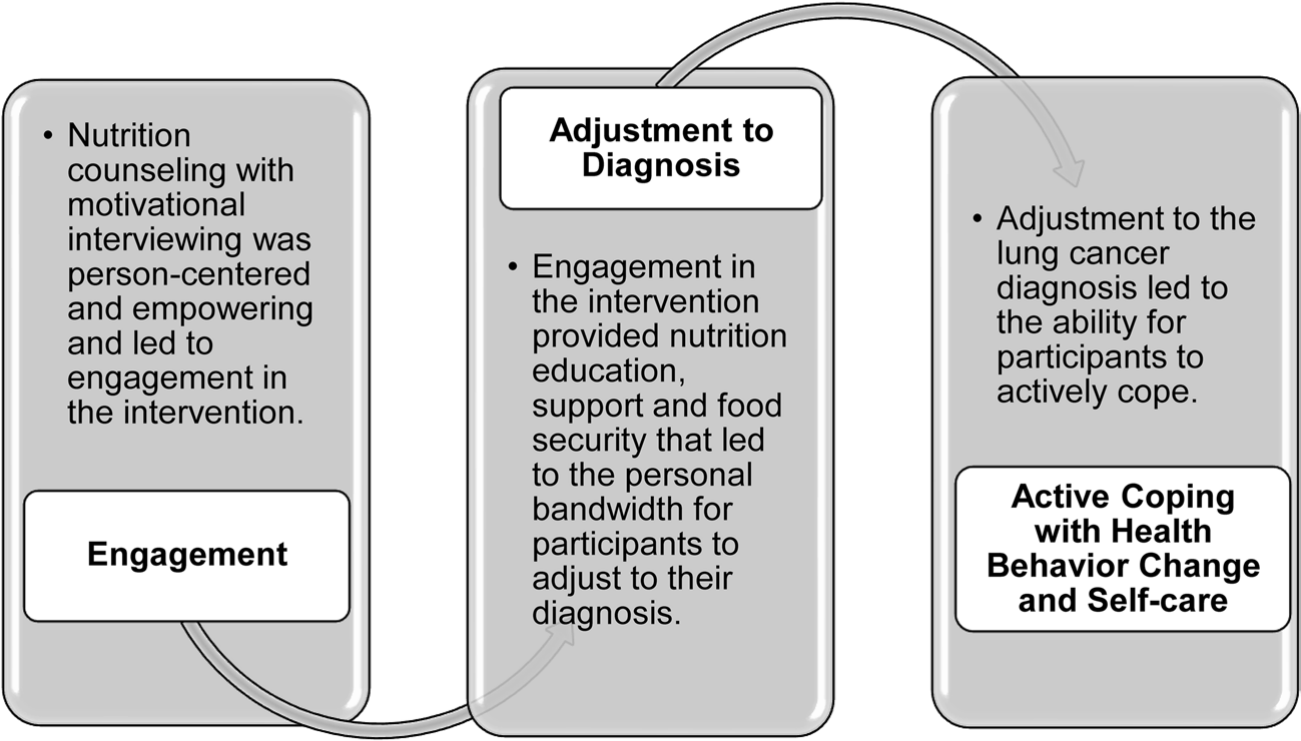
The NutriCare process

The theoretical process that explained how NutriCare worked is based on the patterned relationships we discerned as engaging participants in treatment, adjustment to diagnosis, and active coping and self-care. NutriCare engaged participants in treatment to support their adjustment to a lung cancer diagnosis and treatment which enabled active coping and self-care (Fig. 1).

The overarching outcome of the NutriCare process was that the intervention appeared to buffer some of the adversity related to a lung cancer diagnosis, and this helped participants develop resilience. This quote exemplifies the resilience participants described, “There are just days – you just – you get feeling bad and you think, wow, I don't know if I'm going to fight this any longer or not…but then you decide to do it. And I also think your mental health, a lot of the good nutrition helps. Because you are more alert…sharper.” (F4)

Behavior changes that were adopted appear to lead to post-traumatic growth and resilience. Participants reported regarding the intervention as a positive experience, and that it helped them become physically and mentally equipped with tools to help them cope with the lung cancer diagnosis. These tools helped participants to actively cope with lung cancer and ultimately to become more resilient during the ups and downs of treatment. For example:

“And like I say, [dietitian] through the NutriCare program has taught me different things I can eat and drink to help calm, you know, combat the nausea and you know, just everything else. It's just made me feel a lot better. The NutriCare and the food from [MANNA] and everything else, it just made it easier for me to prepare, or for [caregiver] to prepare for me when I was really sick.” (O5)

Another patient shared how the program allowed them to execute the behaviors they knew were beneficial even when they were not motivated to do so, thus exhibiting resilience. The patient shared, “Unless it’s been a long time since I’ve eaten, I don’t get hungry. But I know that I need to do it, so I sit down and I eat anyway.” (O9)

## Discussion

The purpose of this study was to explore the perceptions and experiences of vulnerable patients with lung cancer enrolled in the intervention arm of the NutriCare study to provide a theoretical explanation for the intervention process. Our findings suggest that home-delivered medically tailored meals and weekly remote nutrition counseling created engagement in treatment which helped with their adjustment to diagnosis leading to active coping and self-care behaviors (Fig. 1).

Most interventions in chronic disease target health behavior change and report on those outcomes, but few elucidate *how* the intervention process worked, particularly from the perspective of the patient [29]. Ongoing research in co-design, user-centered design, and community participatory research underscores the importance of involving end-user (e.g., patients) in intervention development, design, and evaluation, and their perceptions are of paramount importance to ensure relevance, sustainability, and effectiveness [30–32]. Understanding how an intervention worked is important because a theoretical explanation of the process will guide the development of future programs and research. For example, we learned that person-centered motivational interviewing and medically tailored meals are key components for promulgating an adjustment to diagnosis and active coping with self-care.

Furthermore, understanding how this Food is Medicine intervention worked is significant because a cancer diagnosis has complex psychological and physical impacts [33–35]. Patients report feeling overwhelmed by the complexity of care, feeling ill-prepared to manage side effects of treatment, isolated, unsupported, and “out of their depth” [34]. Despite this, cancer patients engaged in NutriCare and other nutrition and lifestyle interventions report creating personal growth since lifestyle changes are challenges that patients can control during a time of uncertainty [33, 35]. This ability to learn and change despite experiencing adversity is remarkable but aligns with transformative learning and social-cognitive transition models where one is challenged to question existing habits for personal growth [36, 37].

Motivational interviewing integrated with nutrition counseling within the NutriCare intervention, appears to have supported patients to question their lifestyle habits in a way that enabled change. Research shows that motivational interviewing is effective or outperforms traditional educational interventions without motivational interviewing for a broad range of diseases [25, 38, 39]. Motivational interviewing begins with developing trust and is predicated on engaging patients, honoring autonomy, identifying intrinsic motivation, and empowering patients to make behavior change to respect their personal values and priorities, akin to shared decision-making. The participants in the NutriCare study engaged in shared decision-making, becoming more purposeful about making lifestyle changes to actively cope with a serious diagnosis, possibly leading to post-traumatic growth and resilience.

Post-traumatic growth usually involves some level of increased appreciation for life, self-care, improved health behaviors, and improved perception of personal strength, making life more meaningful [35, 40]. During aggressive cancer treatments, patients often report feeling overwhelmed by nutrition-related issues such as food availability, complications related to nutrition impact symptoms, and limited, if any, access to registered dietitian nutritionists [34]. These critical gaps in cancer care contribute to the development of malnutrition and associated increased mortality for vulnerable patients [3]. Full integration of food and nutrition into cancer care for vulnerable patients with lung cancer led to participant perceptions of improvements in dietary patterns and physical activity. The participants described various health behaviors used to prevent or mitigate symptoms. Participants reported that these changes positively impacted their quality of life. These findings underscore the importance of nutrition and the need for dietitians on the oncology care team across the cancer continuum.

NutriCare participants reported that the medically tailored meals also contributed to coping and psychological resilience. Coping is defined as means by which patient participants manage the physical and emotional impacts of their diagnosis [41]. A diagnosis of advanced lung cancer is often accompanied by a myriad of psychological stressors in conjunction with a physical symptom burden, a compromised quality of life, and various monumental medical decisions [42]. Depending on the severity of their diagnosis, people with cancer are faced with the decision to endure a mentally and physically demanding treatment course that often results in symptoms and resulting malnutrition, therefore making nutrition care a critical component in the treatment of advanced lung cancer [42].

Psychological resilience is one’s ability to achieve and/or reestablish a stable mental and physical state in response to a stressful life event [35]. Individual characteristics of psychological resilience include actively coping to facilitate the adjustment to a diagnosis through a positive and optimistic disposition, self-efficacy, cognitive flexibility, social support, and a sense of spirituality [35]. The NutriCare nutrition counseling provided social support and created self-efficacy through shared decision-making. The NutriCare participants communicated that the meal delivery made life easier. This provides preliminary evidence that the meal delivery may have created cognitive flexibility by reducing the stress and cognitive load related to finances, food procurement, and food preparation. Theoretically, this created the emotional bandwidth or cognitive flexibility for participants to actively cope and change lifestyle behaviors [43–45].

## Study limitations

To ensure trustworthiness, we provided quotes to illustrate interpretations for representational adequacy and credibility, described the findings in sufficient depth to demonstrate data saturation and transferability, reached consensus on data analysis through team discussions for dependability, but we did not seek participant confirmation of the findings for confirmability [28]. The research team was composed of content and methodological experts, and the graduate students were trained in interviewing and data analysis. As a team, we practiced reflexivity where we discussed our biases and assumptions to guard against influencing the data analysis, but all the team members were registered dietitian nutritionists in training, or oncologists involved with the NutriCare intervention. Finally, we reached data saturation at interview 17 of 20, but most of the participants were living with stage III or IV non-small cell lung cancer. Our findings may have been more nuanced if we interviewed more participants at an early stage of disease or with small cell lung cancer.

## Conclusion

We developed a theoretical explanation about how the NutriCare intervention worked. This process included three linked and propositional categories: engaging in treatment, adjusting to diagnosis, and active coping—resulting in resilience. The person-centered nutrition counseling with motivational interviewing engaged participants in treatment, which helped participants adjust to their diagnosis, leading to active coping through intentional self-care, behavior change, and improved quality of life. Encouraging engagement in treatment may increase treatment effectiveness.

Our findings provide evidence that a nutrition intervention created a pathway for participants to experience post-traumatic growth. Participants developed resilience and changed health behavior to actively cope with lung cancer. Nutrition counseling with motivational interviewing and meals supported individuals’ adjustment to their diagnosis for positive behavior change. Motivational interviewing in nutrition counseling is shown to be effective for creating lifestyle behavior change and our study adds to this evidence.

In the NutriCare study, nutrition counseling with motivational interviewing and meal delivery appear to mitigate the impact of living with lung cancer by supporting participants to actively cope and make lifestyle behavior changes that created resilience and improved their quality of life. While there is some research about patient’s social cognitive transition in cancer, more research is needed to understand the impact different types and dosages of interventions on patient’s ability to adjust [37, 46].

## Data Availability

No datasets were generated or analysed during the current study.
